# Adverse events of video capsule endoscopy over the past two decades: a systematic review and proportion meta-analysis

**DOI:** 10.1186/s12876-020-01491-w

**Published:** 2020-11-02

**Authors:** Yuan-Chen Wang, Jun Pan, Ya-Wei Liu, Feng-Yuan Sun, Yang-Yang Qian, Xi Jiang, Wen-Bin Zou, Ji Xia, Bin Jiang, Nan Ru, Jia-Hui Zhu, En-Qiang Linghu, Zhao-Shen Li, Zhuan Liao

**Affiliations:** 1 National Clinical Research Center for Digestive Diseases, Department of Gastroenterology, Changhai Hospital, 168 Changhai Road, Shanghai, 200433 China; 2 Department of Gastroenterology, The First Medical Center of PLA General Hospital/Chinese PLA Postgraduate Military Medical School, 28 Fuxing Road, Beijing, 100853 China

**Keywords:** Video capsule endoscopy, Adverse events, Systematic review and meta-analysis, Predictors, Temporal-trend

## Abstract

**Background:**

A full spectrum of video capsule endoscopy (VCE) adverse events over the past two decades has not been evaluated. We aimed to determine pooled rates, predictors and temporal-trend of VCE adverse events over the past two decades.

**Methods:**

Systematic search of PubMed and EMBASE for English-language publications reporting VCE adverse events (January 1, 2000 to March 31, 2019). Data were extracted independently by two investigators. Pooled VCE adverse event rates were calculated using the random or fixed model as appropriate. Predictors and temporal-trend of each adverse event were performed by meta-regression analyses.

**Results:**

In total, 402 studies were identified, including 108,079 VCE procedures. Rate of retention, swallow disorder, aspiration, technical failure, and procedural adverse events were 0.73% (95% confidence interval [CI] 0.59–0.89%), 0.75% (95% CI 0.43–1.13%), 0.00% (95% CI 0.00–0.00%), 0.94% (95% CI 0.65–1.28%), 0.67% (95% CI 0.32–1.10%), respectively; incomplete examination rate of esophagus, stomach, small bowel, and colon were 9.05%, 7.69%, 12.08%, 19.19%, respectively. Patency capsule reduced retention rate by 5.04%, whereas known inflammatory bowel disease increased retention rate by 4.29%. Elder was the risk and protective factor for small bowel incomplete examination (0.30%) and swallow disorder (− 0.72%), respectively. Rates of retention and small bowel incomplete examination significantly declined over time (*P* = .0006 and *P* < .0001)..

**Conclusions:**

VCE adverse event rates were generally low, and retention and small bowel incomplete examination rates declined over the past two decades. Patients with known inflammatory bowel disease or elder should be alerted to high risk of retention or small bowel incomplete examination (PROSPERO: CRD42019139595).

## Background

Since its introduction by Iddan et al. [1] in 2000, video capsule endoscopy (VCE) has established itself as a noninvasive diagnostic tool for gastrointestinal diseases over the past two decades. It has become the first-line investigation procedure in small bowel disorder evaluation [2]. Recently, the invention of esophagus capsule endoscopy (ECE) [3, 4], magnetically controlled capsule endoscopy (MCE) [5–7], and colon capsule endoscopy (CCE) [8, 9] widened the range of applications and made VCE available for entire gastrointestinal tract examination.

Although VCE has been widely used, potential VCE adverse events could happen and deserved consideration. Retention, the most noticed adverse event that may lead to acute small bowel obstruction and usually required surgical intervention. It has been reported of approximately 1.4% in most recent review [10] and varied from 0 to 13% [11–17]. Related systematic reviews reported pooled retention rate of different indications (1.2–2.6% and 2.1–8.2%, respectively) [18, 19]. However, no systematic review or meta-analysis estimated the rate of a full spectrum of VCE adverse events, and predictors of each adverse event have never been evaluated. Additionally, with the advance of technology, VCE adverse event rates tend to decline and an update is warranted. Moreover, previous SRMAs are limited to single VCE type, while several novel diagnostic VCEs have been invented and widely used in clinical practice, adverse event rates of other VCE types are needed.

Herein, we aimed to perform a comprehensive systematic review of the contemporary literature to quantify the rates of all VCE adverse events, assessing the potential predictors of each adverse event and demonstrating whether rates changed over the past 20 years.

## Methods

### Data sources and searches

This systematic review and meta-analysis was conducted and reported in accordance with the Preferred Reporting Items for Systematic Review and Meta-Analysis (PRISMA) guidelines (Additional file 1: Method 1) [20]. The identifier of systematic review registration was PROSPERO (CRD42019139595). We searched PubMed and EMBASE databases for English-language publications on VCE from January 1, 2000 through March 31, 2019 using the keywords related to “capsule endoscopy”, which were based on Medical Subject Headings. Additional studies were identified by manually searching the reference lists of the included studies. Detailed search strategy is available in Additional file 1: Method 2.

### Study selection

Studies reporting adverse events of VCE were included. Exclusion criteria included (1) Case reports or studies with fewer than 50 patients; (2) letters, editorials, correspondences, perspectives, reviews, guidelines, conference abstracts or presentation without formal publication; (3) Animal and in vitro studies, trainee participation; (4) Duplicated publications from the same trial (only the most recent and most extensive data was included); (5) Studies focused on non-VCE (i.e. motility capsule endoscopy, patency capsule endoscopy [PCE] only, BRAVO pH capsule, tethered capsule endoscopy, balloon capsule endoscopy, et al). Studies that performed an initial PCE before VCE to exclude potential small bowel obstruction were included. Three independent reviewers (Y.-C.W., J.P., and Y.-W.L.) selected the abstracts and determine their inclusion. Full texts of the potentially eligible studies were further evaluated whether it contained relevant information.

### Definitions

We defined VCE adverse events as retention, swallow disorder, aspiration, technique failure, procedural adverse events, and incomplete examination of esophagus, stomach, small bowel, and colon. Retention was defined as VCE remaining in the gastrointestinal tract for minimum two weeks and retention confirmed with abdominal radiograph, or if a directed medical, endoscopic or surgical intervention has to be implemented to remove or add its passage [18, 21]. Swallow disorder was defined as patients unable to swallow the VCE, or require endoscopic delivery system assistance [22, 23]. The definition of aspiration was bronchial aspired VCE [24]. Technical failure was defined as malfunction of the equipment, including gaps in recording, short duration of batteries of VCE or recorder, failure to activate VCE, failure to download or upload [25]. Procedural adverse events meant discomfort during VCE examination [26]. Esophagus incomplete examination was defined as no image of Z line was obtained by VCE [27]. Stomach incomplete examination was defined as incomplete visualization of all six landmarks (i.e. Cardia, fundus, body, angulus, antrum, and pylorus) [28]. Small bowel incomplete examination, meaning that VCE failure to reach the caecum during the recording time [18, 29]. Colon incomplete examination was regarded as VCE was not excreted or did not reach the rectum during the recording time [30].

### Data extraction and outcomes assessment

Data were extracted independently by two investigators (Y.-C.W., F.-Y. S.). The characteristics of study (i.e. first author, publication year, study period, study design, area), patient (i.e. Simple size, mean age, male percentage, indications, history), procedure (i.e. total number of VCE, VCE types), and each adverse event (i.e. Type, events number, reasons, and interventions) were independently collected. Patient groups were classified by indications according to the clinical practice guidelines [10], as for the case-controlled studies and randomized controlled trials (RCT), the data of each group was extracted separately.

Our primary outcome was to estimate pooled rate of each VCE adverse event. The secondary outcomes were factors associated with the rate of retention, small bowel incomplete examination, swallow disorder, and procedural adverse events. Time-trend of all VCE adverse events were analyzed to determine whether rates changed over the past two decades.

### Data synthesis and analysis

All statistical analyses were performed using the meta package in R version 3.5.1, and statistical significance was reported when the *P* < .05 unless specified otherwise. The VCE adverse events rates were pooled using *metaprop* command. We applied Freeman-Tukey Double Arcsine transformations since low rates were expected [31]. Heterogeneity was assessed with the *I*^*2*^ statistic, which values of 0%, < 25%, 25–75%, and ≥ 75% denoted no, low, moderate, and high heterogeneity, respectively [32]. According to the degree of heterogeneity, random effects model described by Dersimonian Laird [33] or fixed model was used. Publication bias was assessed mathematically using Egger’s test [34]. Sensitivity analysis were performed by systematically removing each study in turn to explore its effect on each VCE adverse event rate.

Subgroup analysis was done according to different VCE type (ECE, Gastric VCE [GCE], Small Bowel CE [SBCE], CCE, and After PCE). The *metareg* command was used in univariate and multivariate meta-regressions to test the influence of study-level moderators on the rate of retention, small bowel incomplete examination, swallow disorder, and procedural adverse events [35]. Seven moderators were tested including study midpoint period, study design, study region, patient groups, male percentage, mean age, and VCE type. Covariates meeting our significance criterion (*P* ≤ .1) were entered into a multivariate meta-regression model. The study period midpoint and each adverse event rate were meta-regressed to explore which adverse event’s rate have changed over time.

## Results

**Fig. 1:**
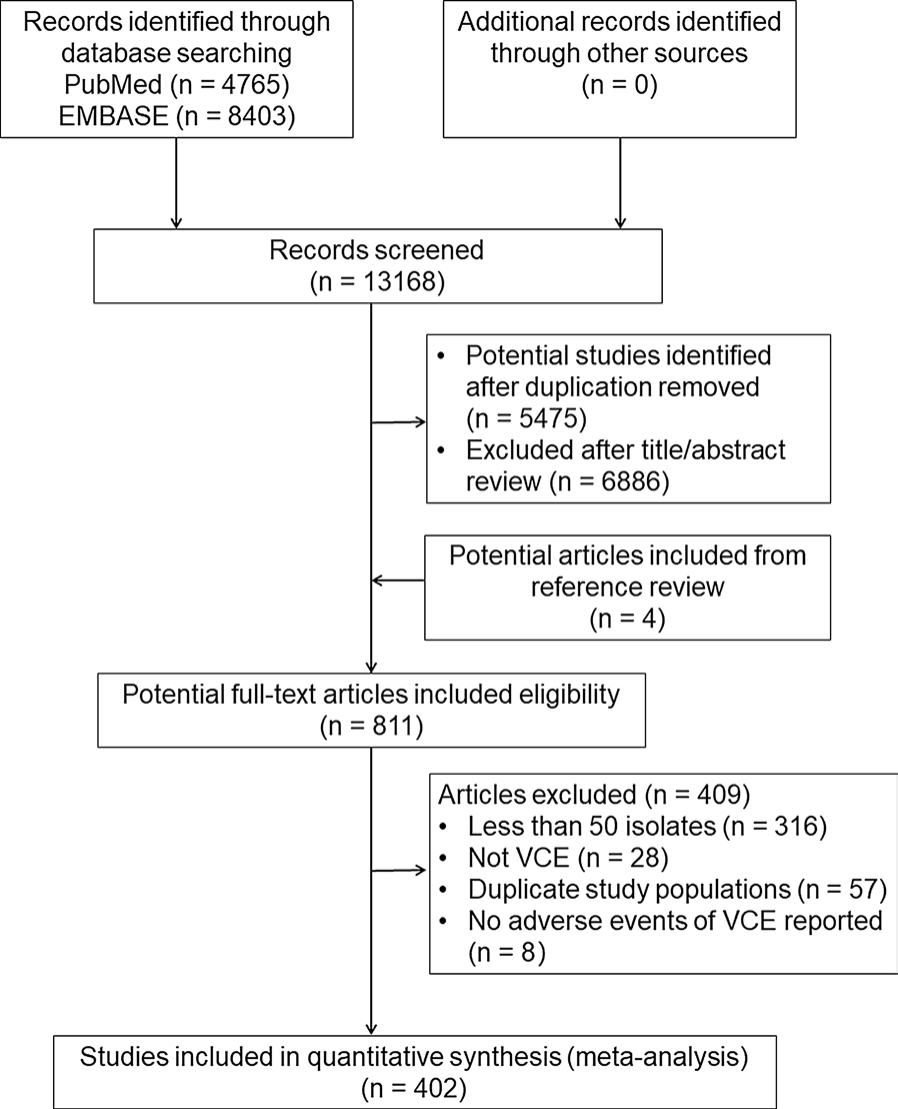
The flowchart of the study selection process

The literature search resulted in 13,168 citations, 811 potentially relevant studies met the eligibility criteria were reviewed in full. After excluding ineligible reports, 402 studies were selected for systematic review and meta-analysis (Fig. 1).

### Study characteristics

The baseline characteristics of the included studies were summarized in Table 1. The final analysis included 108,079 VCE procedures, the SBCE usage rate was predominated (303 studies [75.37%], 91,872 SBCE [85.00%] out of 91,069 patients [84.89%]). As time went on, ECE (study period midpoint, 2008 [range 2005–2012]), CCE (study period midpoint, 2012 [range 2006–2017]), and GCE (study period midpoint, 2014 [range 2004–2017]) had been invented in succession. Most studies were observational designed (360 [89.55%]; including 156 prospective and 204 retrospective studies), forty-two (10.45%) studies were RCT. The studies were conducted mainly in the Europe (n = 172 [42.79%]) and Asia (n = 136 [33.83%]), followed by North America (n = 79 [19.65%]). Fewer studies were conducted in Oceania (n = 8 [1.99%]), multiple continents (n = 3, 0.75%), Latin America (n = 3 [0.75%]), and Africa (n = 1 [0.25%]). The mean age of patients was 52.56 years (range 9.92–73.3 years) and approximately equal sex distribution (mean prevalence of males, 52.51%).

**Table 1:** Characteristics of studies included in meta-analysis

	No. (%)					
	ECE	GCE	SBCE	CCE	After PCE	Overall
Total						
Included studies	26	15	303	43	15	402
Patient, n	2469	5197	91,069	5918	2624	107,277
VCE, n	2473	5197	91,872	5963	2574	108,079
Study characteristics						
Midpoint of study period, mean (range)	2008 (2005–2012)	2014 (2004–2017)	2008 (2001–2018)	2012 (2006–2017)	2011 (2006–2015)	2009 (2001–2018)
Study design						
RCT	3	1	32	6	0	42
Prospective	23	11	81	34	7	156
Retrospective	0	3	190	3	8	204
Region						
Europe	12	4	122	28	6	172
North America	11	0	64	3	1	79
Asia	2	11	105	11	7	136
Oceania	1	0	7	0	0	8
Latin America	0	0	3	0	0	3
Africa	0	0	1	0	0	1
Multiple	0	0	1	1	1	3
Patient characteristics						
Mean age, y	55.03	48.46	53.14	55.21	33.33	52.56
Male sex, %	65.89	57.31	51.16	52.47	51.86	52.51
Patient groups						
Population-based	23	13	155	37	6	234
Known IBD	0	0	23	3	7	33
OGIB	2	2	91	1	0	96
Abdominal pain or diarrhea	0	0	8	0	0	8
Suspected IBD	0	0	9	0	0	9
NSAIDs users	0	0	8	0	0	8
Mixed high-risk group	0	0	6	0	2	8
Suspected tumor	1	0	3	2	0	6

VCE, video capsule endoscopy; ECE, esophagus capsule endoscopy; GCE, gastral capsule endoscopy; SBCE, small bowel capsule endoscopy; CCE, colon capsule endoscopy; PCE, patency capsule endoscopy; IBD, inflammatory bowel disease; OGIB, obscure gastrointestinal bleeding; NSAIDs, non-steroidal anti-inflammatory drugs

### Overall VCE adverse event rate and publication bias

The pooled rate of retention, swallow disorder, aspiration, technical failure, and procedural adverse events were 0.73% (1096/86,742; 95% confidence interval [CI] 0.59–0.89%; 289 studies), 0.75% (426/37,270; 95% CI 0.43–1.13%; 155 studies), 0.00% (5/23,449; 95% CI 0.00–0.00%; 86 studies), 0.94% (396/37,297; 95% CI 0.65–1.28%; 146 studies), 0.67% (198/18,317; 95% CI 0.32–1.10%; 108 studies), respectively; the esophagus, stomach, small bowel, and colon incomplete examination pooled rate were 9.05% (112/924; 95% CI 3.14–17.33%; 12 studies), 7.69% (103/4027; 95% CI 2.45–15.21%; 12 studies), 12.08% (9902/68,091; 95% CI 10.89–13.32%; 278 studies), and 19.19% (874/4483; 95% CI 14.06–24.88%; 37 studies), respectively (Table 2 and Additional file 1: Figs. S1 to S6). The characteristics of VCE technical failures were summarized in Additional file 1: Table S1.

**Table 2:** Pooled rate of each VCE adverse events and subgroup analysis based on VCE types

VCE type and the rate of adverse events	VCE types											
	ECE			GCE			SBCE			CCE		
	AER, % (95% CI)	Study, n	Events/VCE, n	AER, % (95% CI)	Study, n	Events/VCE, n	AER, % (95% CI)	Study, n	Events/VCE, n	AER, % (95% CI)	Study, n	Events/VCE, n
Retention	0.11 (0.00–0.46)	18	8/1622	0.39 (0.19–0.64)	13	40/5072	0.93 (0.75–1.12)	217	1017/74115	0.26 (0.00–0.77)	26	22/3432
Esophagus IE	9.05 (2.43–18.88)	10	103/825	9.06 (3.95–15.76)	2	9/99	–	–		–	–	
Stomach IE	14.47 (3.65–30.35)	4	52/326	5.27 (0.36–14.24)	6	40/3494	4.18 (0.00–13.89)	2	11/207	–	–	
Small bowel IE	–	–		13.91 (3.40–29.51)	5	174/863	13.17 (11.88–14.50)	245	9507/62956	3.99 (2.51–5.75)	21	136/3004
Colon IE	–	–		–	–		–	–		19.19 (14.06–24.88)	37	874/4483
Swallow disorder	1.76 (0.88–2.86)	19	39/1741	0.00 (0.00–0.02)	11	2/4451	0.85 (0.41–1.42)	95	336/26021	0.04 (0.00–0.18)	27	12/4427
Aspiration	0.00 (0.00–0.82)	3	0/220	0.00 (0.00–0.27)	7	0/641	0.00 (0.00–0.00)	57	5/19866	0.00 (0.00–0.00)	18	0/2671
Technical failure	1.16 (0.52–1.98)	14	24/1596	0.20 (0.00–0.98)	8	6/843	0.83 (0.51–1.21)	98	300/31068	1.76 (0.76–3.06)	26	66/3790
Procedural adverse events	6.48 (2.65–11.65)	19	133/1695	0.09 (0.00–0.93)	8	7/4098	0.00 (0.00–0.05)	59	20/9378	0.81 (0.15–1.80)	21	33/3040

VCE type and the rate of adverse events	VCE types				Overall				
	After PCE			*P* value between groups	AER, % (95% CI)	Study, n	Events/VCE, n	*I*^*2*,^ %	Egger test *P* value
	AER, % (95% CI)	Study, n	Events/VCE, n						
Retention	0.09 (0.00–0.34)	15	9/2501	<*.0001*	0.73 (0.59–0.89)	289	1096/86742	70.6	.6063
Esophagus IE	–	–		.9523	9.05 (3.14–17.33)	12	112/924	92.8	.7632
Stomach IE	–	–		.3350	7.69 (2.45–15.21)	12	103/4027	95.5	*.0017*
Small bowel IE	3.79 (0.12–11.08)	7	85/1268	< *.0001*	12.08 (10.89–13.32)	278	9902/68091	95.6	.1315
Colon IE	–	–		–	19.19 (14.06–24.88)	37	874/4483	95.2	.1393
Swallow disorder	7.80 (0.00–26.93)	3	37/630	< *.0001*	0.75 (0.43–1.13)	155	426/37270	89.7	*.0000*
Aspiration	0.00 (0.00; 3.34)	1	0/51	.5201	0.00 (0.00–0.00)	86	5/23449	0.00	*.0000*
Technical failure	–	–		.0707	0.94 (0.65–1.28)	146	396/37297	83.4	*.0000*
Procedural adverse events	4.72 (1.35–9.73)	1	5/106	< *.0001*	0.67 (0.32–1.10)	108	198/18317	84.1	.*0000*

AER, adverse event rate; CI, confidence interval; IE, incomplete examination. *P* value between groups < .05 indicated a significant difference of adverse event rate between various VCE types. Egger test *P* value < .05 showed an obvious publication bias

The Egger’s test did not indicate the existence of obvious publication bias for retention rate (*P* = .6063), incomplete examination rate of esophagus (*P* = .7632), small bowel (*P* = .1315), and colon (*P* = .1393), while for the rate of stomach incomplete examination (*P* = .0017), swallow disorder (*P* < .0001), aspiration (*P* < .0001), technical failure (*P* < .0001), and procedural adverse events (*P* < .0001) showed significant asymmetry (Table 2). The effect estimated from the sensitivity analysis showed little change (Additional file 1: Fig. S7).

### Subgroup analysis and meta-regression analysis

#### Factors and predictors associated with retention rate

Subgroup analysis according to VCE type indicated SBCE associated with higher retention rate (1017/74,115; 0.93%, 95% CI 0.75–1.12%). Univariate meta-regression analysis suggested that study period midpoint, patient groups, and VCE type were eligible for inclusion in multivariate analysis. The after PCE (coefficient = − 5.04%, 95% CI − 8.75% to − 1.33%, *P* = .0077) and known IBD (coefficient = 4.29%, 95% CI 1.46–7.12%, *P* = .0029), remained significant (Table 3).

**Table 3:** Meta-regression of VCE retention rate

	Univariate meta-regression			Multivariate meta-regression^a^		
	Coefficient (95% CI)	Studies, n	*P *value	Coefficient (95% CI)	Studies, n	*P* value
Study period midpoint^b^	− 0.34 (− 0.53 to − 0.14)	245	*.0006*	− 0.24 (− 0.46 to − 0.02)	245	*.0348*
Study design						
RCT	Reference	25	Reference	–	–	–
Prospective	− 0.49 (− 3.36 to 2.38)	115	.7382	–	–	–
Retrospective	1.05 (− 1.71 to 3.81)	149	.4559	–	–	–
Study region						
Asia	Reference	106	Reference	–	–	–
Europe	− 1.31 (− 2.93 to 0.31)	120	.1125	–	–	–
North America	− 0.64 (− 2.71 to 1.43)	50	.5464	–	–	–
Oceania	0.52 (− 4.56 to 5.61)	7	.8402	–	–	–
Multiple	− 2.31 (− 6.85 to 2.24)	6	.3195	–	–	–
Patient groups						
Population-based	Reference	160	Reference	Reference	160	Reference
Known IBD^b^	3.07 (0.53 to 5.61)	30	*.0176*	4.29 (1.46 to 7.12)	30	*.0029*
OGIB	1.43 (− 0.30 to 3.17)	67	.1058	–	–	–
Abdominal pain or diarrhea	4.19 (− 0.73 to 9.11)	6	.0948	3.14 (− 1.75 to 8.04)	6	.2084
Suspected IBD	1.49 (− 3.31 to 6.28)	9	.5432	–	–	–
NSAIDs users	− 4.62 (− 10.16 to 0.92)	5	.1020	–	–	–
Mixed high-risk group	− 0.90 (− 5.40 to 3.61)	7	.6966	–	–	–
Suspected tumor	− 3.12 (− 8.83 to 2.59)	5	.2847		–	
Male	− 3.37 (− 9.99 to 3.25)	261	.3186	–	–	–
Mean age	− 0.02 (− 0.09 to 0.04)	219	.5238	–	–	–
VCE type						
SBCE	Reference	217	Reference	Reference	217	Reference
After PCE^b^	− 4.43 (− 7.67 to − 1.18)	15	*.0074*	− 5.04 (− 8.75 to − 1.33)	15	*.0077*
CCE	− 2.98 (− 5.68 to − 0.29)	26	*.0297*	− 2.60 (− 6.06 to 0.86)	26	.1401
ECE	− 3.66 (− 7.02 to − 0.31)	18	*.0323*	− 3.90 (− 9.05 to 1.26)	18	.1390
GCE	− 2.82 (− 6.31 to 0.68)	13	.1139	–	–	–

^a ^Multivariate meta-regression was performed when the univariate meta-regression *P* value was ≤ .1

^b ^Moderators had a significant effect on VCE retention rate

#### Factors and predictors associated with small bowel incomplete examination rate

Subgroup analysis of VCE type showed small bowel incomplete examination rate was markedly lower in CCE and after PCE group (136/3004, 3.99% [95% CI 2.51–5.75%] and 85/1268, 3.79% [95% CI 0.12–11.08%], respectively; *P* < .0001). Univariate meta-regression analysis showed that study area, patient groups, mean age, and VCE type were significant predictors, and multivariate meta-regression showed that multiple continents (coefficient = − 19.57%, 95% CI − 38.64% to − 0.49%, *P* = .0444), mean age (coefficient = 0.30%, 95% CI 0.10–0.49%, *P* = .0031), and CCE (coefficient = − 10.76%, 95% CI − 19.50% to − 2.02%, *P* = .0158) had a significant effect on small bowel incomplete examination rate (Additional file 1: Table S2).

#### Factors and predictors associated with swallow disorder rate

In VCE subgroup analysis, swallow disorder rate was highest in after PCE group (37/630, 7.80%, 95% CI 0.00–26.93%). In univariate meta-regression analysis, retrospective design, study region, known IBD group, mean age, and after PCE were predictor of swallow disorder rate. Multivariate meta-regression showed that Europe (coefficient = 4.01%, 95% CI 0.02–8.00%, *P* = .0486), North America (coefficient = 7.51%, 95% CI 2.26–12.76%, *P* = .0051), Oceania (coefficient = 20.80%, 95% CI 8.01–33.58%, *P* = .0014), known IBD (coefficient = − 16.49%, 95% CI − 24.68 to − 8.30%, *P* < .0001), and mean age (coefficient = − 0.72%, 95% CI − 0.89 to − 0.56%, *P* < .0001) significantly associated with swallow disorder rate (Additional file 1: Table S3).

#### Factors and Predictors associated with procedural adverse events rate

The procedural adverse events rate in ECE was significantly higher than other VCE types (133/1695, 6.48%, 95%CI 2.65–11.65%, *P* < .0001) according to VCE subgroup analysis. Univariate meta-regression analysis showed that prospective designed, North America, OGIB group, male, and VCE type were significantly affected procedural adverse events rate. The multivariate analysis showed North America (coefficient = 5.85%, 95% CI 0.34–11.36%, *P* = .0373), male (coefficient = 23.90%, 95% CI 7.56–40.24%, *P* = .0041), and ECE (coefficient = 11.38%, 95% CI 4.37–18.40%, *P* = .0015) were the significant predictors of procedural adverse events rate (Additional file 1: Table S4).

### Reasons and interventions of retention and time-trends of each VCE adverse event rate

The definite reasons for retention were reported in 610 VCEs according to 119 studies (Additional file 1: Fig. S8). Crohn’s disease was the most common retention reason (n = 216, 35.41%). Among the 766 retained capsules, surgery was the most frequently used intervention (n = 352, 45.95%), followed by endoscopically management (n = 199, 25.98%), no intervention (n = 176, 22.98%) and medical therapy (n = 39, 5.09%). Although there was no significant change in time-trend analysis of retention interventions, surgery had a downward trend and other interventions had upward trends (Additional file 1: Fig. S9).

**Fig. 2:**
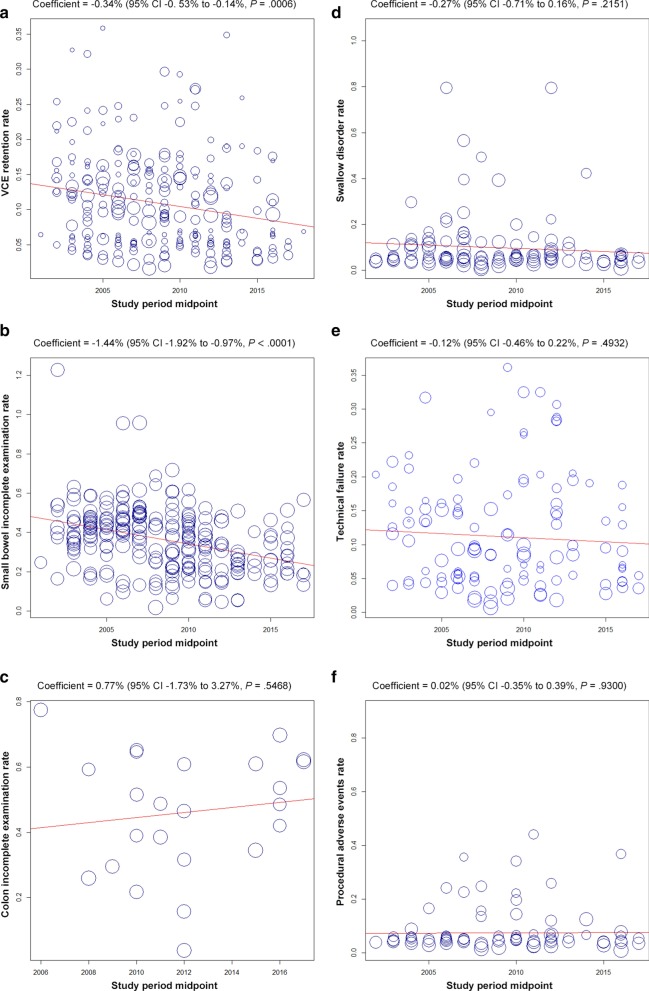
Time-trend analysis of VCE adverse events rates. **a** Retention rate, **b** small bowel incomplete examination rate, **c** colon incomplete examination rate, **d** swallow disorder rate, **e** technical failure rate, **f** procedural adverse events rate

The retention rate (coefficient = − 0.34%, 95% CI − 0.53 to − 0.14%, *P* = .0006) and small bowel incomplete examination rate (coefficient = − 1.44, 95% CI − 1.92 to − 0.97, *P* < .0001) decreased significantly over the years (Fig. 2). The small bowel incomplete examination rate of SBCE significantly declined over time (*P* < .0001), while the rate of CCE unchanged (*P* = .6815) (Additional file 1: Fig. S10). The stomach and esophagus incomplete examination rate were not analyzed because there was an insufficient number of studies.

## Discussion

To our knowledge, this is the first meta-analysis to report pooled rates of each VCE adverse event, assess the predictors and provide time-trend analysis over the past two decades. The results demonstrated that VCE is a safe procedure worldwide with low rate of adverse events. The risk for retention should be assumed in patients with known IBD, which is a contraindication unless intestinal patency is proven, best by the passage of an intact PCE. Elder age is the risk or protect factor for small bowel incomplete examination or swallow disorder. In addition, the rate of retention and small bowel incomplete examination were declined over the years.

Retention is the most focused adverse event since retained capsules may cause partial or complete gastrointestinal obstruction, which limits wider utilization of VCE. The known IBD, with underlying inflammatory strictures [36], results in a higher retention rate, the most recent study by Pasha et al. indicated the retention rate of established Crohn’s disease was 4.63% (95% CI 3.42–6.25%) [37], and our study indicated known IBD increases retention rate by 4.29%. However, compared with previous study [18], we detected a lower pooled retention rate of 0.73%. This could be attributed to the usage of PCE, which predicts small bowel strictures in high-retention risk patients [38, 39]. As showed in the results, retention rate of after PCE group was 0.09% in subgroup analysis, and PCE significantly decreased retention rate by 5.04% in multivariate meta-regression. These findings confirm that performing an initial PCE before VCE in patients with a high-risk of retention, represented by the known IBD, is useful to avoid retention [40, 41]. It is noteworthy that not all patients undergoing VCE should be offered a patency capsule since several complications have been reported, including small bowel obstruction [42] and perforation [43]. Surgery is frequently performed for retained capsules in the early years [44]; however, for asymptomatic patients or with slight abdominal pain, later studies reported more favorable clinical outcomes using endoscopic methods or medical treatment [45, 46]. Our time-trend meta-regressions also showed decreasing trend for surgery and increasing trends for non-surgical management.

As VCE is usually swallowed in standing position, the esophageal transit time is very short due to gravity function, resulting in few images taken and causing esophagus incomplete examination. Right supine position [27], acquired image from both ends of VCE [47], increased frame rate [48], and stringed VCE [49, 50] were studied to overcome gravity effect and improved Z-line visualization. The invention of MCE provided a more viable approach for gastric examinations [6]. Since MCE cannot perform flushing and suctioning, visualization may be impaired by the presence of bubbles and mucus. The investigators have used detergents in gastric preparation, while the fundus still poor visualized [51, 52]. Because battery life is limited, incomplete examination of small bowel and colon could easily occur (12.08% and 19.19%, respectively). In this meta-analysis, age was an independent predictor affected small bowel incomplete examination rate, which is similar with the previous study of Girelli et al. [53]. Small bowel incomplete examination rate declined over the past two decades, which may contributed by various investigations, such as prolonged battery life span in newer capsule generation [54, 55] or reduced gastric transit time (GTT). CCE was introduced with prolonged battery life, and we confirmed a significant relationship between CCE and lower small bowel incomplete examination rate. The methods included real-time viewer [56], administered prokinetic agents (such as metoclopramide [57, 58], mosapride [59], and erythromycin [60]), endoscopically placement [61, 62], and magnetic steering [63] can be used to improve the likelihood of a complete small bowel examination in routine clinical practice.

Since the VCE was introduced, it has been proven useful for many indications across a wide age range, the youngest child used VCE was only 8 months of age [64]. However, young age was an independent predictor significantly associated with higher swallow disorder rate. In one series, 63 of 83 children < 8 years old required AdvanCE™ placement device to deliver the VCE into the duodenum [65]. In this study, 0.75% patients were unable to swallow the capsule. It’s notable that, the capsule aspiration is an adverse event relating to swallowing disorder. Although very rare (5/23,449), case reports described it may cause life threatening acute respiration distress, and over half of patients required bronchoscopy intervention after capsule aspiration [24, 66, 67]. Aging, neurological or swallowing disorder and patients with a weak or absent cough are high risk for VCE aspiration [68]. Fortunately, the patients included in this meta-analysis had no respiratory distress, and the problem can resolved quickly by spontaneously coughing [23, 25, 53, 69].

The overall technical failures rate is significantly lower than previous study by Rondonotti et al. (0.67% vs 8.59%) [25], this reflects the immature of early capsule and software prototypes. During VCE examination, male and ECE were significantly increased procedural adverse events rate. In esophagus examination, the use of string attachment could be able to prevent VCE rapid and unpredictable transmission, allowing controllable movement and real-time visualization. However, the retrieval of the capsule caused discomfort, usually lead to nausea and vomiting [3]. The detachable technique in string VCE avoid this problem, and comfort assessment was better than previous reports [50].

To date, this work is the largest overview including over 100,000 VCE procedures from 402 literatures. Our study has several strengths. First, compared with previous studies, this is the first systematic review and meta-analysis comprehensively summarized the full spectrum of VCE adverse events, ranging from retention, swallow disorder, aspiration, technique failure, procedural adverse events, and incomplete examination, demonstrating low adverse event rates and safe clinical application over its two decades of use. Second, we performed meta-regression to determine the predictors of each adverse event, and identified new risk or protective factor (age) for small bowel incomplete examination or swallow disorder. Third, this is the first study providing temporal changes of VCE adverse event rates. The retention and small bowel incomplete examination rates in this study were lower than previous systematic review [18] (0.73% vs 1.4%, and 12.08% vs 16.5%, respectively), and our time-trend analysis indicated rates of those adverse events declined over the past two decades, encouraging continued efforts to achieve and maintain safety targets in VCE practice.

There are several limitations. First, there were obvious heterogeneity in most VCE adverse event rates, and Egger test indicated potential publication bias for stomach incomplete examination rate, swallow disorder rate, aspiration rate, technical failure rate, and procedural adverse events rate, which may have compromised the precision of our study. Second, exclusion of studies with fewer than 50 patients may introduced selection bias to this analysis, the rate of rare VCE adverse events such as capsule aspiration may underestimated. Last, meta-regression analysis was conducted on the level of the studies, and the characteristics of studies, individual patients, and VCE could not be retrieved to identify other risk factors.

## Conclusions

This systematic review and meta-analysis comprehensively summarized the full spectrum of VCE adverse events, demonstrating low adverse event rates and safe clinical application. Retention and small bowel incomplete examination rates declined over the past two decades. Patients with known inflammatory bowel disease or elder should be alerted to high risk of retention or small bowel incomplete examination. Future clinical practice and research will benefit from this knowledge and potential adverse events would be prevented.

## Supplementary information

**Additional file 1**. Supplementary online content.

## Data Availability

The datasets during and/or analyzed during the current study available from the corresponding author on reasonable request.

## Abbreviations

VCE: Video capsule endoscopy
ECE: Esophagus capsule endoscopy
MCE: Magnetically controlled capsule endoscopy
CCE: Colon capsule endoscopy
PRISMA: Preferred Reporting Items for Systematic Review and Meta-Analysis
PCE: Patency capsule endoscopy
RCT: Randomized controlled trials
GCE: Gastric capsule endoscopy
SBCE: Small bowel capsule endoscopy
OGIB: Obscure gastrointestinal bleeding
GTT: Gastric transit time
IBD: Inflammatory bowel disease
NSAIDs: Nonsteroidal anti-inflammatory drugs
IE: Incomplete examination
CI: Confidence interval
AER: Adverse event rate
