# Supplementary material for: Adverse events of video capsule endoscopy over the past two decades: a systematic review and proportion meta-analysis
Source: BMC Gastroenterol. 2020 Nov 2;20:364. doi: 10.1186/s12876-020-01491-w (PMC7607645; doi:10.1186/s12876-020-01491-w)
Supplement: Supplementary file 1 — Additional file 1. Supplementary online content. [file 12876_2020_1491_MOESM1_ESM.docx]

Supplementary Online Content

Additional file 1: Method 1. PRISMA checklist of this systematic review and meta-analysis.

Additional file 1: Method 2. Search strategy used in the current systematic review and meta-analysis.

Additional file 1: Table S1. Characteristics of VCE technical failures.

Additional file 1: Table S2. Meta-regression of small bowel incomplete examination rate.

Additional file 1: Table S3. Meta-regression of swallow disorder rate.

Additional file 1: Table S4. Meta-regression of procedural adverse events rate.

Additional file 1: Fig. S1. Forest plot showing the pooled rate of VCE retention.

Additional file 1: Fig. S2. Forest plot showing the pooled rate of incomplete examination. (a) Esophagus incomplete examination rate;(b) Stomach incomplete examination rate; (c) Small bowel incomplete examination rate; (d) Colon incomplete examination rate.

Additional file 1: Fig. S3. Forest plot showing the pooled rate of swallow disorder.

Additional file 1: Fig. S4. Forest plot showing the pooled rate of VCE aspiration.

Additional file 1: Fig. S5. Forest plot showing the pooled rate of technical failure.

Additional file 1: Fig. S6. Forest plot showing the pooled rate of procedural adverse events.

Additional file 1: Fig. S7. Sensitivity analysis of (a) Stomach incomplete examination rate; (b) Swallow disorder rate; (c) Technical failure rate; (d) Procedural adverse events rate.

Additional file 1: Fig. S8. Definite reasons for VCE retention.

Additional file 1: Fig. S9. Time-trend analysis of retention interventions. (a) Surgery; (b) Endoscopically management; (c) Medical therapy; (d) No intervention.

Additional file 1: Fig. S10. Time-trend analysis of small bowel incomplete examination rates by (a) SBCE, and (b) CCE.

Additional file 1: Method 1. PRISMA checklist of this systematic review and meta-analysis.

| **Section/topic** | **#** | **Checklist item** | **Reported on page #** |
| --- | --- | --- | --- |
| **TITLE** | | |  |
| Title | 1 | Identify the report as a systematic review, meta-analysis, or both. | 1 |
| **ABSTRACT** | | |  |
| Structured summary | 2 | Provide a structured summary including, as applicable: background; objectives; data sources; study eligibility criteria, participants, and interventions; study appraisal and synthesis methods; results; limitations; conclusions and implications of key findings; systematic review registration number. | 3-4 |
| **INTRODUCTION** | | |  |
| Rationale | 3 | Describe the rationale for the review in the context of what is already known. | 5-6 |
| Objectives | 4 | Provide an explicit statement of questions being addressed with reference to participants, interventions, comparisons, outcomes, and study design (PICOS). | 6 |
| **METHODS** | | |  |
| Protocol and registration | 5 | Indicate if a review protocol exists, if and where it can be accessed (e.g., Web address), and, if available, provide registration information including registration number. | 7 |
| Eligibility criteria | 6 | Specify study characteristics (e.g., PICOS, length of follow-up) and report characteristics (e.g., years considered, language, publication status) used as criteria for eligibility, giving rationale. | 7 |
| Information sources | 7 | Describe all information sources (e.g., databases with dates of coverage, contact with study authors to identify additional studies) in the search and date last searched. | 7 |
| Search | 8 | Present full electronic search strategy for at least one database, including any limits used, such that it could be repeated. | 7 |
| Study selection | 9 | State the process for selecting studies (i.e., screening, eligibility, included in systematic review, and, if applicable, included in the meta-analysis). | 7-8 |
| Data collection process | 10 | Describe method of data extraction from reports (e.g., piloted forms, independently, in duplicate) and any processes for obtaining and confirming data from investigators. | 8-9 |
| Data items | 11 | List and define all variables for which data were sought (e.g., PICOS, funding sources) and any assumptions and simplifications made. | 9 |
| Risk of bias in individual studies | 12 | Describe methods used for assessing risk of bias of individual studies (including specification of whether this was done at the study or outcome level), and how this information is to be used in any data synthesis. | NA |
| Summary measures | 13 | State the principal summary measures (e.g., risk ratio, difference in means). | 9-10 |
| Synthesis of results | 14 | Describe the methods of handling data and combining results of studies, if done, including measures of consistency (e.g., I^2^) for each meta-analysis. | 10 |

| **Section/topic** | **#** | **Checklist item** | **Reported on page #** |
| --- | --- | --- | --- |
| Risk of bias across studies | 15 | Specify any assessment of risk of bias that may affect the cumulative evidence (e.g., publication bias, selective reporting within studies). | 10 |
| Additional analyses | 16 | Describe methods of additional analyses (e.g., sensitivity or subgroup analyses, meta-regression), if done, indicating which were pre-specified. | 10-11 |
| **RESULTS** | | |  |
| Study selection | 17 | Give numbers of studies screened, assessed for eligibility, and included in the review, with reasons for exclusions at each stage, ideally with a flow diagram. | 12 |
| Study characteristics | 18 | For each study, present characteristics for which data were extracted (e.g., study size, PICOS, follow-up period) and provide the citations. | 12-13 |
| Risk of bias within studies | 19 | Present data on risk of bias of each study and, if available, any outcome level assessment (see item 12). | NA |
| Results of individual studies | 20 | For all outcomes considered (benefits or harms), present, for each study: (a) simple summary data for each intervention group (b) effect estimates and confidence intervals, ideally with a forest plot. | 13 |
| Synthesis of results | 21 | Present results of each meta-analysis done, including confidence intervals and measures of consistency. | 13 |
| Risk of bias across studies | 22 | Present results of any assessment of risk of bias across studies (see Item 15). | 13-14 |
| Additional analysis | 23 | Give results of additional analyses, if done (e.g., sensitivity or subgroup analyses, meta-regression [see Item 16]). | 14-17 |
| **DISCUSSION** | | |  |
| Summary of evidence | 24 | Summarize the main findings including the strength of evidence for each main outcome; consider their relevance to key groups (e.g., healthcare providers, users, and policy makers). | 18-23 |
| Limitations | 25 | Discuss limitations at study and outcome level (e.g., risk of bias), and at review-level (e.g., incomplete retrieval of identified research, reporting bias). | 23 |
| Conclusions | 26 | Provide a general interpretation of the results in the context of other evidence, and implications for future research. | 24 |
| **FUNDING** | | |  |
| Funding | 27 | Describe sources of funding for the systematic review and other support (e.g., supply of data); role of funders for the systematic review. | 26-27 |

*From:*  Moher D, Liberati A, Tetzlaff J, Altman DG, The PRISMA Group (2009). Preferred Reporting Items for Systematic Reviews and Meta-Analyses: The PRISMA Statement. PLoS Med 6(6): e1000097. doi:10.1371/journal.pmed1000097

For more information, visit: **www.prisma-statement.org**.

Additional file 1: Method 2. Search strategy used in the current systematic review and meta-analysis

PubMed

((((((((((((((("Capsule Endoscopy"[Mesh]) OR capsule endoscopies) OR endoscopies, capsule) OR endoscopy, capsule) OR wireless capsule endoscopy) OR wireless capsule endoscopies) OR capsule endoscopies, wireless) OR capsule endoscopy, wireless) OR endoscopies, wireless capsule) OR endoscopy, wireless capsule) OR video capsule endoscopy) OR capsule endoscopies, video) OR capsule endoscopy, video) OR endoscopies, video capsule) OR endoscopy, video capsule) OR video capsule endoscopies Filters: Publication date from 2000/01/01 to 2019/03/31

EMBASE

('capsule endoscopy'/exp OR 'capsule endoscopies' OR 'endoscopies, capsule' OR 'endoscopy, capsule' OR 'wireless capsule endoscopy' OR 'wireless capsule endoscopies' OR 'capsule endoscopies, wireless' OR 'capsule endoscopy, wireless' OR 'endoscopies, wireless capsule' OR 'endoscopy, wireless capsule' OR 'video capsule endoscopy' OR 'capsule endoscopies, video' OR 'capsule endoscopy, video' OR 'endoscopies, video capsule' OR 'endoscopy, video capsule' OR 'video capsule endoscopies') AND [1-1-2000]/sd NOT [1-4-2019]/sd

Additional file 1: Table S1. Characteristics of VCE technical failures.

| VCE technical failures | Study, n | Events, n | Total technical events, n | % |
| --- | --- | --- | --- | --- |
| Gap in recording | 26 | 91 | 155 | 58.7% |
| Gap in transmission | 13 | 28 | 62 | 45.2% |
| Short duration of batteries of VCE or recorder | 19 | 103 | 172 | 59.9% |
| Failure to activate VCE | 6 | 20 | 80 | 25.0% |
| Failure of download or upload | 5 | 15 | 95 | 15.8% |

Additional file 1: Table S2. Meta-regression of small bowel incomplete examination rate

|  | Univariate meta-regression | | |  | Multivariate meta-regression ^a^ | | |
| --- | --- | --- | --- | --- | --- | --- | --- |
|  | Coefficient (95% CI) | Studies, n | P value |  | Coefficient (95% CI) | Studies, n | P value |
| Midpoint of study period ^b^ | -1.44 (-1.92 to -0.97) | 246 | **<.0001** |  | -1.40 (-1.97 to -0.83) | 246 | **<.0001** |
| Study design |  |  |  |  |  |  |  |
| RCT | Reference | 29 | Reference |  | - | - | - |
| Prospective | -0.83 (-7.44 to 5.77) | 83 | .8045 |  | - | - | - |
| Retrospective | 4.06 (-2.09 to 10.20) | 166 | .1954 |  | - | - | - |
| Study region |  |  |  |  |  |  |  |
| Asia | Reference | 96 | Reference |  | Reference | 96 | Reference |
| Europe | -1.37 (-5.56 to 2.81) | 116 | .5201 |  | - | - | - |
| North America | 5.46 (0.35 to 10.58) | 55 | **.0363** |  | 0.43 (-5.49 to 6.35) | 55 | .8871 |
| Oceania | 1.42 (-10.53 to 13.37) | 7 | .8157 |  | - | - | - |
| Multiple ^b^ | -16.32 (-33.69 to 1.06) | 3 | .0657 |  | -19.57 (-38.64 to -0.49) | 3 | **.0444** |
| Patients group |  |  |  |  |  |  |  |
| Population-based | Reference | 151 | Reference |  | Reference | 151 | Reference |
| Known IBD | -2.56 (-9.59 to 4.47) | 22 | .4754 |  | - | - | - |
| OGIB | 2.06 (-2.20 to 6.32) | 78 | .3423 |  | - | - | - |
| Abdominal pain or diarrhea | 5.02 (-6.09 to 16.13) | 8 | .3759 |  | - | - | - |
| Suspected IBD | -6.75 (-18.81 to 5.32) | 7 | .2730 |  | - | - | - |
| NSAIDs users | -15.20 (-29.07 to -1.33) | 5 | **.0318** |  | -14.24 (-30.31 to 1.82) | 5 | .0823 |
| Mixed high-risk group | 14.39 (-0.97 to 29.76) | 4 | .0664 |  | 5.60 (-10.04 to 21.24) | 4 | .4828 |
| Suspected tumor | -5.99 (-23.88 to 11.90) | 3 | .5116 |  | - | - |  |
| Male | 5.51 (-12.43 to 23.45) | 251 | .5472 |  | - | - | - |
| Mean age ^b^ | 0.23 (0.06 to 0.40) | 206 | **.0086** |  | 0.30 (0.10 to 0.49) | 206 | **.0031** |
| CE type |  |  |  |  |  |  |  |
| SBCE | Reference | 245 | Reference |  | Reference | 245 | Reference |
| After PCE | -16.84 (-28.18 to -5.49) | 7 | **.0036** |  | -11.51 (-25.62 to 2.61) | 7 | .1101 |
| CCE ^b^ | -16.15 (-22.99 to -9.32) | 21 | **<.0001** |  | -10.76 (-19.50 to -2.02) | 21 | **.0158** |
| GCE | 1.20 (-12.42 to 14.81) | 5 | .8633 |  | - | - | - |

^a^ Multivariate meta-regression was performed when the univariate meta-regression *P* value was ≤ .1.

^b^ Moderators had a significant effect on VCE retention rate.

Additional file 1: Table S3. Meta-regression of swallow disorder rate

|  | Univariate meta-regression | | |  | Multivariate meta-regression **^a^** | | |
| --- | --- | --- | --- | --- | --- | --- | --- |
|  | Coefficient (95% CI) | Studies, n | P value |  | Coefficient (95% CI) | Studies, n | P value |
| Study Midyear | -0.27 (-0.71 to 0.16) | 122 | .2151 |  | - | - | - |
| Study design |  |  |  |  |  |  |  |
| RCT | Reference | 18 | Reference |  | Reference | 18 | Reference |
| Prospective | 4.81 (-0.77 to 10.40) | 78 | .0913 |  | 0.82 (5.23 to 6.87) | 78 | .7906 |
| Retrospective | 6.12 (0.42 to 11.83) | 59 | **.0353** |  | 4.39 (-1.42 to 10.20) | 59 | .1383 |
| Study region |  |  |  |  |  |  |  |
| Asia | Reference | 53 | Reference |  | Reference | 53 | Reference |
| Europe ^b^ | 4.60 (0.67 to 8.52) | 68 | **.0217** |  | 4.01 (0.02 to 8.00) | 68 | **.0486** |
| North America ^b^ | 5.10 (0.09 to 10.11) | 28 | **.0462** |  | 7.51 (2.26 to 12.76) | 28 | **.0051** |
| Oceania ^b^ | 19.64 (8.52 to 30.76) | 4 | **.0005** |  | 20.80 (8.01 to 33.58) | 4 | **.0014** |
| Patients group |  |  |  |  |  |  |  |
| Population-based | Reference | 110 | Reference |  | Reference | 110 | Reference |
| Known IBD ^b^ | 6.76 (-1.01 to 14.54) | 8 | .0882 |  | -16.49 (-24.68 to -8.30) | 8 | **<.0001** |
| OGIB | -1.91 (-6.86 to 3.04) | 22 | .4494 |  |  | - |  |
| Abdominal pain or diarrhea | -5.41 (-16.05 to 5.22) | 4 | .3186 |  |  | - |  |
| Mixed high-risk group | -6.10 (-18.21 to 6.01) | 3 | .3238 |  |  | - |  |
| Suspected tumor | -1.59 (-12.62 to 9.45) | 4 | .7782 |  |  | - |  |
| Male | 7.65 (-7.98 to 23.29) | 142 | .5137 |  | - | - | - |
| Mean age ^b^ | -0.56 (-0.70 to -0.42) | 121 | **<.0001** |  | -0.72 (-0.89 to -0.56) | 121 | **<.0001** |
| CE type |  |  |  |  |  |  |  |
| SBCE | Reference | 95 | Reference |  | Reference | 95 | Reference |
| After PCE | 16.70 (4.51 to 28.89) | 3 | **.0072** |  | 10.35 (-3.65 to 24.36) | 3 | .1473 |
| CCE | -3.97 (-8.62 to 0.67) | 27 | .0935 |  | - | - | - |
| ECE | 4.33 (-1.13 to 9.80) | 19 | .1203 |  | - | - | - |
| GCE | -5.32 (-12.10 to 1.45) | 11 | .1235 |  | -5.93 (-12.84 to 0.97) | 11 | .0922 |

^a^ Multivariate meta-regression was performed when the univariate meta-regression *P* value was ≤ .1.

^b^ Moderators had a significant effect on VCE retention rate.

Additional file 1: Table S4. Meta-regression of procedural adverse events rate

|  | Univariate meta-regression | | |  | Multivariate meta-regression **^a^** | | |
| --- | --- | --- | --- | --- | --- | --- | --- |
|  | Coefficient (95% CI) | Studies, n | P value |  | Coefficient (95% CI) | Studies, n | P value |
| Study Midyear | 0.02 (-0.35 to 0.39) | 81 | .9300 | - | - | - | - |
| Study design |  | - |  |  |  | - |  |
| RCT | Reference | 15 | Reference |  | Reference | 15 | Reference |
| Prospective | 5.49 (0.06 to 10.91) | 62 | **.0474** | - | 3.49 (-2.07 to 9.06) | 62 | .2189 |
| Retrospective | -3.34 (-9.21 to 2.53) | 31 | .2647 | - | - | - | - |
| Study region |  | - |  |  |  | - |  |
| Asia | Reference | 31 | Reference |  | Reference | 31 | Reference |
| Europe | 1.74 (-3.00 to 6.48) | 47 | .4720 | - | - | - | - |
| North America ^b^ | 9.56 (4.12 to 15.00) | 26 | **.0006** | - | 5.85 (0.34 to 11.36) | 26 | **.0373** |
| Patients group |  |  |  |  |  |  |  |
| Population-based | Reference | 72 | Reference |  | Reference | 72 | Reference |
| Known IBD | -6.40 (-15.85 to 3.04) | 5 | .1838 |  | - | - | - |
| OGIB | -6.69 (-12.06 to -1.33) | 19 | **.0145** | - | -1.27 (-6.69 to 4.15) | 19 | .6458 |
| Suspected IBD | -5.27 (-17.59 to 7.06) | 3 | .4023 |  |  | - |  |
| Mixed high-risk group | -2.39 (-14.48 to 9.69) | 3 | .6980 |  |  | - |  |
| Male ^b^ | 41.30 (26.70 to 55.91) | 101 | **<.0001** | - | 23.90 (7.56 to 40.24) | 101 | **.0041** |
| Mean age | 0.16 (-0.06 to 0.38) | 84 | .1631 | - | - | - | - |
| CE type |  | - |  |  |  | - |  |
| SBCE | Reference | 59 | Reference |  | Reference | 59 | Reference |
| CCE | 5.87 (1.50 to 10.24) | 21 | **.0084** | - | 3.52 (-2.54 to 9.57) | 21 | .2551 |
| ECE ^b^ | 20.41 (15.77 to 25.05) | 19 | **<.0001** | - | 11.38 (4.37 to 18.40) | 19 | **.0015** |
| GCE | 1.38 (-4.95 to 7.70) | 8 | .6695 | - | - | - | - |

^a^ Multivariate meta-regression was performed when the univariate meta-regression *P* value was ≤ .1.

^b^ Moderators had a significant effect on VCE retention rate.
